# Preface: International Commission on Trichinellosis recommendations for the detection and control of *Trichinella*

**DOI:** 10.1016/j.fawpar.2019.e00063

**Published:** 2019-08-14

**Authors:** Brad Scandrett, Dante Zarlenga

**Affiliations:** a Canadian Food Inspection Agency, Centre for Food-borne and Animal Parasitology, 116 Veterinary Road, Saskatoon, SK S7N 2R3, Canada; b United States Department of Agriculture, Agricultural Research Service, Animal Parasitic Diseases Lab, B1180 BARC-East, Beltsville, MD, USA

Trichinellosis, caused by nematodes of the genus *Trichinella*, has a worldwide distribution and a long history of causing human disease. As a zoonosis, trichinellosis has had a major economic impact on the global trade of pork. It was one of the first zoonoses for which regulated veterinary controls were established, in the late 1880s. It is also an excellent example of an infectious disease of relevance to both humans and animals that can be prevented and controlled through a ‘One Health’ approach.

When the parasite was first discovered in the mid-1800s and for many years thereafter, it was believed that this genus was monospecific. Within the last 50 years, our knowledge has expanded greatly, particularly in the fields of molecular biology, epidemiology and control. Consequently, the taxonomy of *Trichinella* has evolved accordingly. This was advanced, in part, by the formation of a centralized database and specimen bank at the International *Trichinella* Reference (ITR) Center in Rome, Italy, and by the advent of modern techniques of diagnosis. The ITR database, comprising more than 7500 isolates to date, has facilitated the assembly and comparison of epidemiological, biological and genetic data that have permitted the identification of at least 9 different species and 3 distinct genotypes whose classifications remain in flux.

The importance of this disease brought together key researchers, regulators and others with backgrounds in the medical and veterinary sciences and with interests including more fundamental aspects of the biology and ecology of *Trichinella* and trichinellosis. The idea for a global commission on trichinellosis was first entertained in 1958 in Budapest, Hungary at the Hungarian Meeting of Parasitologists. Two years later, the International Commission on Trichinellosis (ICT) was formally established at a meeting on *Trichinella* sponsored by the Polish Parasitological Society in Warsaw, Poland and, with few exceptions, has held meetings every 4 years ever since.

The mission of the ICT is to exchange and enhance knowledge on all aspects of the genus *Trichinella*, including the infections caused by these parasites in animals and humans. To this end, ICT has charged itself with providing guidance, recommendations and responsible opinions on the parasite, the disease, human exposure, and control. Developing the recommendations included in this special issue not only encompassed the expertise of ICT scientists, but of national and international organizations and authorities such as the World Organization for Animal Health, World Health Organization, and the Food and Agriculture Organization. These recommendations have found application locally and globally through the development of regulations and guidelines on *Trichinella* control.

Joint action by ICT members has resulted in the five new recommendations published in this special issue of Food and Waterborne Parasitology that reflect the current state of knowledge for: 1) Pre-Harvest Control; 2) Post-Harvest Control; 3) Serology; 4) Genotyping *Trichinella* Muscle Stage Larvae; and 5) Quality Assurance in Digestion Testing for *Trichinella.* Similar information pertaining to these recommendations is also available on the ICT website (http://www.trichinellosis.org). The editors thank the authors of the recommendations published herein, and all those who contributed to this endeavor, including incumbent ICT president Joke van der Giessen, Isabelle Vallée, and the external reviewers who provided valuable edits and comments to further improve the final articles.

